# Pandemic (H1N1) 2009 in Skunks, Canada

**DOI:** 10.3201/eid1606.100352

**Published:** 2010-06

**Authors:** Ann P. Britton, Ken R. Sojonky, Andrea P. Scouras, Julie J. Bidulka

**Affiliations:** Animal Health Centre, British Columbia Ministry of Agriculture and Lands, Abbotsford, British Columbia, Canada

**Keywords:** Pandemic, influenza virus A, pandemic (H1N1) 2009 virus, striped skunks, pneumonia, viruses, influenza, Mephitidae, letter

**To the Editor**: In March 2009, a novel influenza virus A (H1N1) emerged in Mexico, and, because of widespread human-to-human transmission, a global pandemic was declared in June 2009 (*1*). Although most cases have involved humans, pandemic (H1N1) 2009 has sporadically infected swine and turkeys and has also been reported in a small number of pet ferrets, cats, and captive cheetahs, and in a dog (*2*). Many of these animals were cared for by persons who experienced influenza-like illness and the owner of 1 cat who died had confirmed pandemic (H1N1) 2009 respiratory disease before the cat became ill, which suggests probable human-to animal-transmission of the virus (*2*).

During mid-December 2009–mid-January 2010, eight striped skunks (*Mephitis mephitis*) died on a mink farm near Vancouver, British Columbia, Canada. On January 12, 2010, two of the skunks were brought to the Animal Health Centre in Abbotsford, British Columbia, for postmortem examination. One skunk exhibited purulent nasal exudates. In both skunks, investigators observed splenomegaly and severe pneumonia, characterized by heavy, dark red to purple, lung lobes involving >70% of the lung field. Microscopic examination showed moderate rhinitis and severe bronchopneumonia with intralesional bacteria, areas of interstitial pneumonia, and occasional nematode larvae. Also observed were splenic extramedullary hematopoiesis, plasmacytosis of both lymph nodes and spleen, and mild plasmacytic glomerulonephritis with proteinuria.

Routine bacteriologic culture of lung showed heavy growth of *Streptococcus dysgaslactiae* subsp. *equisimilis*, *Staphylococcus aureus,* and *Hafnia alvei*. That death was caused by uncomplicated mixed bacterial bronchopneumonia in 2 (and possibly up to 8) adult skunks over a 6-week period was considered unlikely. The presence of lungworm was considered incidental. However, the areas of interstitial pneumonia suggested that a primary viral pathogen was likely.

Molecular testing was conducted initially on fresh lung, liver, kidney, and spleen for canine distemper virus and, subsequently, for influenza A virus. The splenic and nodal plasmacytosis and plasmacytic glomerulonephritis also prompted testing for Aleutian disease virus (ADV). Organ samples were negative for canine distemper virus and positive for ADV.

Detection of influenza A virus nucleoprotein and matrix genes and hemagglutinin and neuraminidase typing was performed with real-time reverse transcription–PCR. Organ samples were positive for pandemic (H1N1) 2009, which was confirmed by sequence analysis of DNA fragments obtained in the hemagglutinin, neuraminidase, and matrix gene testing.

Primary viral interstitial pneumonia is frequently complicated by opportunistic bacterial bronchopneumonia and influenza virus A infection has been shown to predispose to pulmonary bacterial toxicity (*3*). Thus, we concluded that primary pandemic (H1N1) 2009 interstitial pneumonia had predisposed the 2 skunks to mixed bacterial bronchopneumonia and death. The skunks were also infected with ADV, presumably as a result of viral shedding by the minks, which are known to be ADV carriers. Striped skunks can be experimentally infected with ADV, and antibodies to ADV have been detected in wild skunks (*4*). Although ADV does not cause pneumonia (*4*), co-infection with ADV and influenza A virus is associated with higher mortality rates in minks with respiratory disease (*5*). Thus, ADV co-infection may have contributed to the severity of the pneumonia and the death of the skunks.

The source of the pandemic (H1N1) 2009 virus is unclear. Nasal discharge was also observed in many of the minks, which suggests that they had a respiratory viral infection. However, no diagnostic workup was undertaken. Although severe outbreaks of interstitial pneumonia on mink farms can occur (*6*), most natural influenza A virus infections in minks are either mild or asymptomatic (*5*). Thus, the minks may also have been infected with pandemic (H1N1) 2009. Many of the pandemic (H1N1) 2009 infections reported in animals are believed to have been the result of exposure to infected humans (*2*). Workers on the mink farm did not experience influenza-like illness. However, humans with asymptomatic pandemic (H1N1) 2009 infection may have transmitted it to the mink. Because the skunks visited the mink farm daily, transmission of pandemic (H1N1) 2009 from humans to minks to skunks is a possibility.

In view of the detection of pandemic (H1N1) 2009 virus in 2 striped skunks with fatal pneumonia, this species should now be regarded as a potential source of influenza A virus. Wild animals participate in the transmission of influenza A viruses between species, and the presence of wildlife on farms is known to be a risk factor for infection of poultry (*7*). Similar to raccoons, skunks express both α2,3 and α2,6 sialic acid receptors for avian and human influenza viruses in the respiratory tract (M. Shrenzel, San Diego Zoo, pers. comm.), which is believed to create the opportunity for mixed influenza infections with potential for genetic reassortment (*8*). Skunks, like raccoons, are highly mobile animals with large home ranges in rural and urban areas, which provides numerous opportunities for influenza A virus exposure and transmission to poultry, livestock, pets, and, ultimately, humans. The inclusion of striped skunks in wildlife influenza surveillance programs may be warranted.
